# Proportional Correlation Between Systemic Inflammation Response Index and Gastric Cancer Recurrence Time: A Retrospective Study

**DOI:** 10.3390/cancers17091415

**Published:** 2025-04-23

**Authors:** Kyungryun In, Sunhyung Kang, Hyunseok Lee, Hyuksoo Eun, Heeseok Moon, Eaumseok Lee, Seokhyun Kim, Jaekyu Sung, Byungseok Lee

**Affiliations:** Division of Gastroenterology, Department of Internal Medicine, Chungnam National University Hospital, Chungnam National University School of Medicine, Daejeon 35015, Republic of Korea; kyungryun.in@gmail.com (K.I.); macmaster@cnuh.co.kr (H.L.); hyuksoo@cnuh.co.kr (H.E.); mhs1357@cnuh.co.kr (H.M.); leeusgi@cnuh.co.kr (E.L.); midoctor@cnuh.co.kr (S.K.); jksung69@cnuh.co.kr (J.S.); gie001@cnuh.co.kr (B.L.)

**Keywords:** stomach neoplasms, recurrence, prognosis, adjuvant chemotherapy

## Abstract

Background: Disease recurrence is the primary cause of death in patients with gastric cancer who have undergone complete surgical resection. No prognostic factors for recurrence, other than the Tumor, Node, and Metastasis stage, have been established. However, recurrence rates differ even within the same Tumor, Node, and Metastasis stage. Therefore, we aimed to develop a new prognostic confidence measure for gastric cancer recurrence and demonstrate its practical utility. Methods: This was a retrospective study based on the medical records of the Chungnam National University Hospital, Republic of Korea. We enrolled patients diagnosed with stage II/III gastric cancer who underwent complete surgical resection and adjuvant chemotherapy over the past 12 years. The association between seven variables, including the systemic inflammation response index (SIRI) and gastric cancer recurrence, was analyzed. Results: A total of 296 patients were enrolled in this study. Although other factors did not exhibit significant correlations, SIRI showed a significant positive correlation with gastric cancer recurrence risk, confirmed through Cox regression testing (hazard ratio, 1.231; 95% confidence interval, 1.04–1.45). Linear regression analysis revealed a significant association between higher SIRI values and shorter recurrence time (*p* = 0.044; β = −0.225). Conclusions: In this study, other than SIRI, effective prognostic factors related to gastric cancer recurrence were not verified, thus indicating SIRI as a potential independent prognostic factor.

## Simple Summary

Gastric cancer often returns even after successful surgery and chemotherapy, making it important to find ways to predict which patients are at higher risk. Current prediction tools mostly rely on cancer staging, but many patients with the same stage still show different outcomes. In this study, we explored whether a blood-based marker called the Systemic Inflammation Response Index (SIRI), which reflects the body’s overall inflammatory status, can help predict gastric cancer recurrence. We found that patients with higher SIRI levels were more likely to experience cancer recurrence and that their cancer returned sooner. This suggests that SIRI could serve as a simple, cost-effective tool to identify high-risk patients, allowing for more personalized follow-up and treatment plans. These findings could help the medical community better understand the role of inflammation in cancer and improve post-surgical care for patients with gastric cancer.

## 1. Introduction

Although the incidence and mortality rates of gastric cancer are decreasing owing to medical advances, it remains one of the cancers with the highest incidence and mortality rates worldwide, especially in East Asia, including the Republic of Korea [1,2]. The standard treatment for stage II and III gastric cancer is surgical gastrectomy and lymph node (D2) dissection [3]. To improve survival outcomes and reduce the risk of recurrence, adjuvant chemotherapy is administered postoperatively [4,5]. In recent years, neoadjuvant chemotherapy (NAC) has been increasingly explored as a therapeutic strategy in patients with locally advanced gastric cancer [6,7]. Alongside this, studies have also investigated the potential survival benefits of D2 plus lymphadenectomy, particularly in patients with bulky nodal or para-aortic metastases following NAC [8]. While NAC is widely implemented before surgery in Western countries, its adoption in East Asia remains more conservative [9]. The 2022 Korean Gastric Cancer Association (KGCA) guidelines suggest that NAC may be “considered” in select patients [10]; however, its use is still limited in real-world clinical practice due to the lack of insurance coverage and institutional variation.

The main cause of death in patients with gastric cancer who undergo complete surgical resection is cancer recurrence, which occurs despite adjuvant chemotherapy administration [11]. The recurrence rate postoperatively is variable, at 20–60% [12,13]. Therefore, accurately predicting the risk of recurrence in individual patients is essential not only for improving gastric cancer survival outcomes but also for enabling preventive strategies and personalized treatment planning. Studies have reported on prognostic factors of gastric cancer recurrence; however, the utility of prognostic factors other than the tumor, node, and metastasis (TNM) stage remains unclear. Even when adjuvant chemotherapy is administered for the same TNM stage, some patients experience recurrence, indicating the influence of other factors in addition to the TNM stage. Gastric cancer has high intra-tumor heterogeneity, and even if patients diagnosed at the same stage are treated, there may be differences in the incidence of resistance to anticancer drugs and recurrence rates [14,15]. Therefore, this study aimed to identify factors other than the TNM stage that can be used to predict gastric cancer recurrence.

The study was initially designed to explore multiple clinicopathological and inflammation-related prognostic factors, with the ultimate goal of developing a comprehensive risk stratification model. Among these, particular attention was given to the Systemic Inflammation Response Index (SIRI)—a systemic inflammation marker calculated from peripheral neutrophil, monocyte, and lymphocyte counts—as a candidate biomarker [16]. Systemic inflammation is increasingly recognized as a driver of tumor progression [17,18,19], and inflammation-based markers such as the neutrophil-to-lymphocyte ratio (NLR) and platelet-to-lymphocyte ratio (PLR) have been proposed as prognostic tools in several malignancies [20,21,22,23,24].

Accordingly, the primary endpoint of this study was to evaluate whether preoperative SIRI levels are independently associated with recurrence-free survival (RFS) in patients with stage II and III gastric cancer who underwent curative gastrectomy. By identifying the prognostic value of SIRI, this study seeks to contribute to more personalized postoperative surveillance and management strategies in gastric cancer care.

## 2. Materials and Methods

### 2.1. Study Population

This retrospective study was conducted on data from patients diagnosed with gastric cancer and treated with adjuvant chemotherapy after gastrectomy at a tertiary medical center in the Republic of Korea, with data collected between 1 January 2010 and 31 December 2022. Patient data were recorded and retrieved from the electronic medical records of Chungnam National University Hospital (Daejeon, Republic of Korea). The inclusion criteria were: (i) patients aged ≥ 18 years; (ii) diagnosis of stage II/III gastric cancer based on the American Joint Committee on Cancer 8th edition staging system; (iii) complete surgical resection (R0 resection); and (iv) postoperative adjuvant chemotherapy. The exclusion criteria were no available surgical pathology results from our hospital; non-performance of R0 resection; diagnosis of pathologic TNM stage I or IV; no postoperative adjuvant chemotherapy; diagnosis of other cancers; and history of previous chemotherapy or radiation treatment. Neoadjuvant chemotherapy was excluded based on the reasons outlined in the Introduction, including its limited clinical use in Korea and potential impact on systemic inflammatory markers, which were central to our analysis. Of the initially identified patients, two had undergone NAC and were excluded to preserve the homogeneity of the study population and ensure consistency in the timing of biomarker assessment.

Patients were followed up through regular outpatient visits, with surveillance imaging and laboratory tests conducted every 6 to 12 months. Recurrence events were confirmed via imaging studies or biopsy when clinically indicated.

All procedures complied with the ethical standards of the appropriate committees on human experimentation (institutional and national) and the Declaration of Helsinki of 1964 and its later versions. The retrospective study protocol was approved by the Institutional Review Board of Chungnam National University Hospital (approval number: 2023-06-037), and the requirement for written consent was waived based on the retrospective design.

### 2.2. Prognostic Factors

To select predictive factors that showed an association with gastric cancer recurrence, in addition to the TNM stage, we selected factors that have been reported to be related to gastric cancer prognosis. In addition to the SIRI (neutrophil count × monocyte count/lymphocyte count) [16], an inflammatory marker obtained using a peripheral blood test, the following prognostic biomarkers, which are included in the majority of gastric cancer pathology findings at our hospital, were investigated: P53 [25,26], E-cadherin [27,28,29], human epidermal growth factor receptor 2 (HER2; using immunohistochemistry) [30,31,32], Lauren classification [33] (a histological classification system), Epstein–Barr virus (EBV; using in situ hybridization) [34,35]; and microsatellite instability (MSI) status (using DNA polymerase chain reaction) [36,37,38].

Using immunohistochemistry, P53 was classified as “overexpressed” if samples showed >10% nuclear-stained cancer cells and as “wild type” if they showed < 10% [39,40]. E-cadherin was described as “not decreased” when the level was preserved and “decreased” when it was reduced or undetected. HER2 was classified as “negative” for 0/1+ and “positive” for 2+/3+, according to immunoreactivity [41]. The Lauren classification was denoted as “intestinal”, “diffuse”, or “mixed type” according to histopathological findings. EBV status was classified as “negative” or “positive”, depending on whether it was detected. The MSI status was classified as “high” for a high MSI degree and “low” for a low degree or stable MSI. SIRI was calculated using the results of the last peripheral blood test performed before gastric cancer resection surgery.

### 2.3. Statistical Analysis

Statistical analyses were performed using SPSS (version 29.0; IBM Corp., Armonk, NY, USA). Nominal variables were analyzed using the chi-squared (χ^2^) test; for continuous variables, cut-off values were obtained using receiver operating characteristic (ROC) curves, converted into nominal variables, and analyzed using χ^2^ tests. To determine whether SIRI increases the recurrence risk regardless of the TNM stage, we compared gastric cancer recurrence rates between high- and low-SIRI groups at the same TNM stage (cut-off, 2.1), using the χ^2^ test and Kaplan–Meier analysis. Cox regression and linear tests were performed using the product. Linear regression analysis was performed to determine the relationship between SIRI and the recurrence time. Statistical significance was set at a two-sided *p*-value < 0.05.

### 2.4. Study Endpoints

The primary endpoint of this study was the prognostic utility of SIRI as an independent marker for the risk of gastric cancer recurrence. The secondary endpoint was the correlation between SIRI values and the timing of recurrence.

### 2.5. Data Availability

The datasets generated and analyzed during the current study are not publicly available because of the privacy of the research participants but are available from the corresponding author upon reasonable request.

## 3. Results

**Figure 1:**
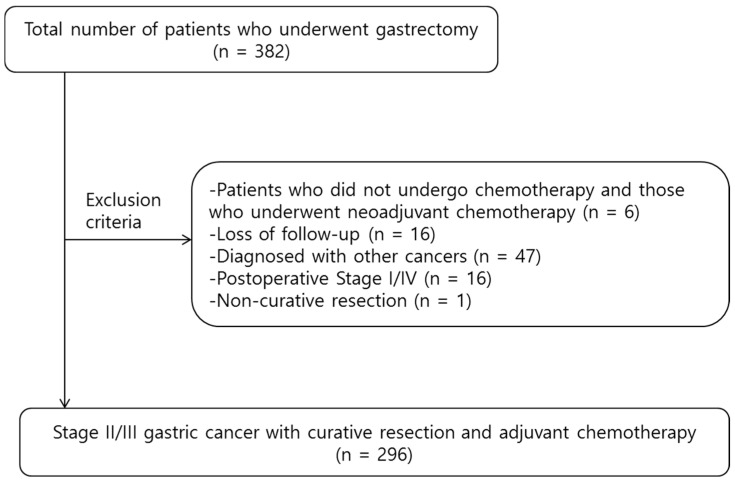
Study flow diagram.

A total of 382 patients were diagnosed with gastric cancer and underwent gastrectomy at Chungnam National University Hospital during the study period. Among them, six patients were excluded for either having received neoadjuvant chemotherapy or not receiving postoperative adjuvant chemotherapy, 16 patients were lost to follow-up, 47 patients had concurrent diagnoses of other cancers, 16 patients had a postoperative pathologic stage of I or IV, and one patient did not achieve R0 resection. After applying these criteria, 296 patients were included. Figure 1 shows a flow chart illustrating the patient selection process. Table 1 summarizes the clinicopathological characteristics of the 296 included patients. The median age was 63.03 years, with 54.1% of patients aged ≤ 65 years and a higher proportion of men (68.2%) than women (31.8%). Regarding tumor location, the majority of tumors were located in the gastric body and antrum (45.6% and 45.3%, respectively), with a smaller percentage found in the cardia (7.1%) and across the entire stomach (2%). Histological differentiation varied, with 52.7% and 46.3% of tumors showing moderate and poor differentiation, respectively. Tumor depth and lymph node involvement were also assessed, showing that 38.9% of patients had T3-stage tumors, and lymph node metastasis was most frequently observed in the N3 category (29.7%). Regarding TNM staging, patients were nearly evenly distributed between stages II (49.7%) and III (50.3%).

During the follow-up period, 82 of 296 patients (27.7%) experienced cancer recurrence. Table 2 presents the results of a χ^2^ analysis examining the association between gastric cancer recurrence and various prognostic factors, including HER2, Lauren classification, p53, SIRI, EBV status, E-cadherin, MSI, and TNM stage. Among these factors, SIRI and TNM stages were significantly associated with cancer recurrence. Specifically, patients with a high SIRI (>2.1) demonstrated a significantly higher recurrence rate than those with a low SIRI (≤2.1; 59% vs. 41%; χ^2^ = 15.35, *p* < 0.001). Regarding the TNM stage, patients with stage III had a significantly higher recurrence rate than those with stage II (44% vs. 12%; χ^2^ = 37.97, *p* < 0.001). However, other factors such as HER2, Lauren classification, p53 expression, EBV status, E-cadherin, and MSI status were not significantly associated with recurrence in this analysis. Although HER2 expression approached statistical significance (*p* = 0.08), it did not reach the conventional threshold.

**Figure 2:**
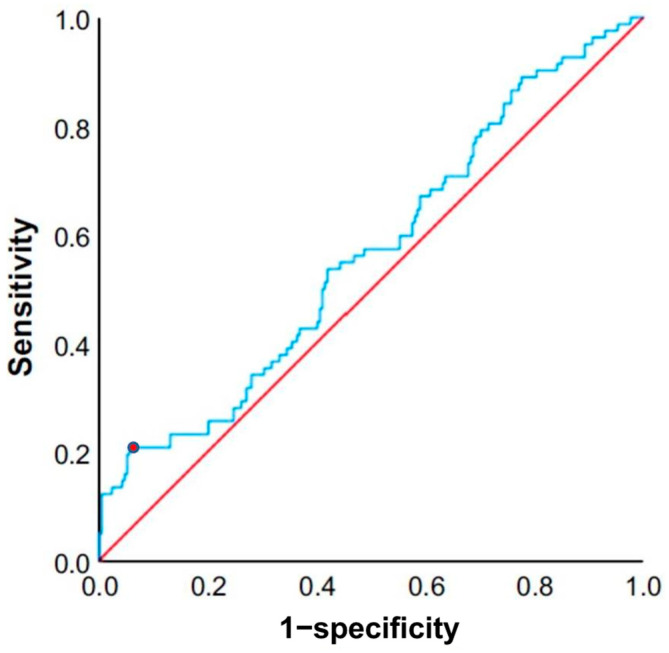
Receiver operating characteristic (ROC) curve for the systemic inflammation response index (SIRI). The cut-off value of SIRI in this study was 2.1 (10^3^/μL), and the area under the curve (AUC) was 0.565. The cut-off point is marked on the curve. The blue line represents the ROC curve for SIRI, and the red diagonal line indicates the reference line (AUC = 0.5).

Figure 2 shows the ROC curve for SIRI in predicting gastric cancer recurrence. The area under the curve (AUC) was 0.565 (95% confidence interval: 0.491–0.638), indicating limited discriminatory performance. The optimal cut-off value of 2.1 (10^3^/μL) was determined using Youden’s index, which identifies the point maximizing the sum of sensitivity and specificity. This cut-off point is marked on the ROC curve. Based on this analysis, patients were classified into high- and low-SIRI groups according to whether their SIRI value was above or below 2.1.

Table 3 shows the association between the SIRI and cancer recurrence within each TNM stage (II and III), using χ^2^ analysis. This analysis was conducted to determine whether SIRI remains a significant predictor of recurrence risk when stratified by TNM stage, which is a well-established prognostic factor in gastric cancer. In patients with TNM stage II, those with a high SIRI (>2.1) had a recurrence rate of 33%, compared with only 10% in the low SIRI group (≤2.1). This difference was statistically significant (χ^2^ = 4.442, *p* = 0.035), indicating that a higher SIRI was associated with an increased risk of recurrence even among patients with similar tumor staging. A similar trend was observed in patients with TNM stage III. Patients with a high SIRI had a significantly higher recurrence rate than those with low SIRI (70% vs. 40%; χ^2^ = 6.535, *p* = 0.0011), suggesting a strong association between elevated SIRI levels and recurrence risk within this more advanced stage group.

**Figure 3:**
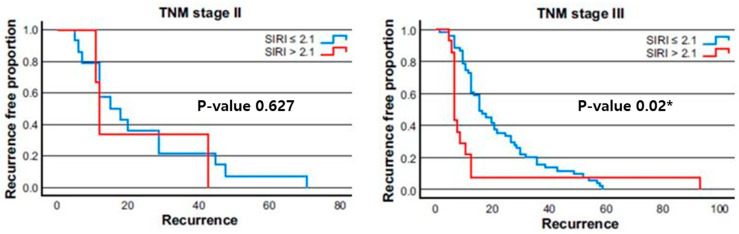
Kaplan–Meier survival curve according to the Tumor, Node, Metastasis (TNM) stage. In TNM stage II, the recurrence-free survival is not significantly different between the high- and low-systemic inflammation response index (SIRI) groups. (*p* = 0.627). In TNM stage III, recurrence was more likely to occur in the high-SIRI group (*p* = 0.02). This result suggests that there may be a greater correlation between SIRI and recurrence in cases with a high TNM stage. An asterisk (*) indicates statistical significance at *p* < 0.05.

Figure 3 shows the Kaplan–Meier recurrence-free survival (RFS) curves stratified by TNM stage (II and III) and SIRI levels (≤2.1 and>2.1). This analysis was performed to explore the impact of systemic inflammation, as measured by SIRI, on RFS within each stage group. In TNM stage II, patients with high SIRI values (>2.1) did not show a statistically significant difference in RFS compared with those with low SIRI values (≤2.1, *p* = 0.627). In contrast, in TNM stage III, a marked difference in RFS was observed between the high-SIRI and low-SIRI groups. Patients with high SIRI values experienced a significantly lower RFS rate than those with low SIRI values (*p* = 0.02). This suggests that for patients with more advanced disease (TNM stage III), elevated SIRI may serve as a more impactful prognostic factor, correlating with a higher recurrence likelihood. These findings imply that the predictive value of SIRI might be more pronounced in higher TNM stages, where the tumor burden and potential for systemic inflammatory response are greater.

To further evaluate the prognostic value of the SIRI as a continuous variable, univariate and multivariate Cox regression analyses were conducted. The univariate Cox regression analysis demonstrated a statistically significant positive correlation between SIRI values and the risk of gastric cancer recurrence, with a hazard ratio of 1.32 (95% CI, 1.127–1.545; *p* < 0.001). This suggests that with each incremental increase in SIRI, recurrence risk increases by approximately 32%. After adjusting for other potential confounding factors in the multivariate analysis, SIRI remained a significant independent predictor of recurrence (hazard ratio, 1.231; 95% CI, 1.04–1.459; *p* = 0.016). This highlights SIRI as a robust marker for predicting recurrence risk even when accounting for other clinical variables.

**Figure 4:**
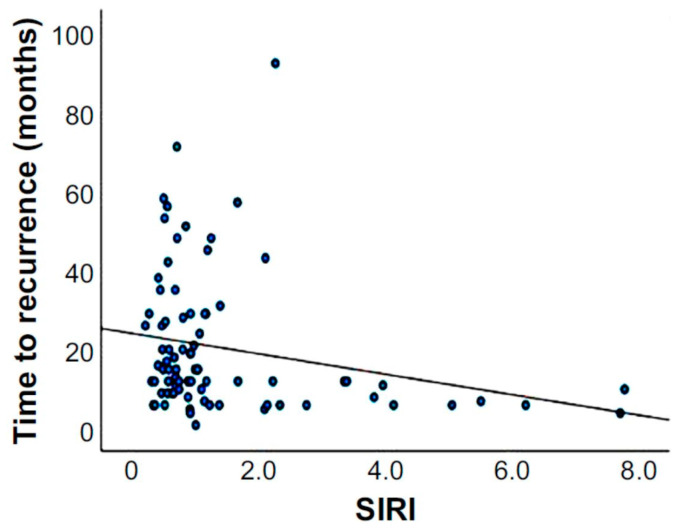
Linear regression analysis for assessing the correlation between SIRI values and recurrence time. The negative correlation was statistically significant (*p* = 0.044; β = −0.225), indicating that the higher the SIRI value, the shorter the time it takes for gastric cancer to recur.

In addition, linear regression analysis was performed to assess the relationship between SIRI and recurrence time (Figure 4), revealing a statistically significant negative correlation (*p* = 0.044; β = −0.225), indicating that higher SIRI values are associated with shorter recurrence time. The regression line in Figure 4 shows that as SIRI increases, recurrence time decreases, underscoring that elevated systemic inflammation may accelerate gastric cancer recurrence.

## 4. Discussion

Postoperative recurrence is a major cause of death in patients with gastric cancer. However, no established prognostic factors, other than TNM, have been identified. Therefore, it is important to identify more prognostic factors for gastric cancer recurrence. During the study design, several pathological factors were considered important; however, none were associated with gastric cancer recurrence. Only SIRI showed significant results other than the TNM stage.

The relationship between inflammation and cancer has been extensively studied over the past 20 years. Immune cells have high plasticity and may show antitumor activity or, conversely, may have pro-tumorigenic activity [42,43,44,45]. In particular, systemic inflammation caused by innate/adaptive immunity is complex and highly variable, causing controversy. However, several epidemiological studies have shown that chronic inflammation causes tumor development. One prospective study showed that the cancer incidence rate in patients with elevated circulating inflammatory markers (for example, C-reactive protein) during routine checkups was more than twice that of the control group [46]. According to a 2018 survey, 42% of adult cancers in North America are caused by correctable risk factors, all of which cause local or systemic inflammation [47]. Large-scale clinical studies have also provided evidence that non-specific inflammation inhibition using non-steroidal anti-inflammatory drugs reduces the incidence and mortality of various cancers [48,49].

Unlike acute inflammation, which promotes antitumor immunity, chronic inflammation is non-resolving [50]. Both acute inflammation and its resolution are complex programmed processes, which, if not controlled, lead to a chronic inflammatory state and immunosuppressive microenvironment through the recruitment of immunosuppressive cells and cytokines [18,19]. As the relationship between inflammation and cancer has been revealed, interest in the tumor microenvironment (TME), which comprises tumor cells and surrounding non-malignant cells, has increased.

A large amount of research is being conducted on the signaling pathways of immune cells and mediators, such as cytokines and chemokines, which make up the TME; recent developments in cancer treatments have focused on related areas [17,51]. The first attempt to classify cancer type in patients with colorectal cancer was based on the immune base rather than the TNM stage. Depending on the degree of cytotoxic T-cell infiltration around the tumor cells, it is classified as a hot, altered, or cold tumor. Cancers with high T-cell infiltration have an antitumor effect and a good prognosis [52,53]. Moreover, the higher expression level of programmed cell death protein 1/programmed cell death ligand 1 (PD-1/PD-L1) in tumor-associated immune cells showed a greater antitumor effect [54]. Accordingly, checkpoint inhibitors have been developed for various cancer types, and the PD-1 inhibitor nivolumab is also used clinically for gastric cancer treatment [55].

Analyzing the local immunity/inflammation of the TME requires tissue and pathological analyses, which are time-consuming and expensive. Moreover, in many cases, a partial, instead of complete, biopsy is performed, which may lead to analysis errors. A clear relationship between systemic and local inflammation has not yet been revealed; however, the immune system of the human body may be related to both local and systemic inflammation. Therefore, studies on systemic inflammatory markers are ongoing. Many studies have reported on various types of systemic inflammatory markers such as neutrophil–lymphocyte, platelet–lymphocyte, and lymphocyte–monocyte ratios and systemic immune-inflammation index in several cancer types [20,21,22,23,24]. Among them, SIRI is an index calculated from the number of neutrophils, monocytes, and lymphocytes in peripheral blood, which is also known to be associated with poor prognosis in various carcinoma types [56]. A high SIRI value indicates an increase in blood neutrophil and monocyte levels and a decrease in lymphocyte levels, which is related to tumor progression, infiltration, and metastasis [57,58,59]. SIRI includes neutrophils and monocytes, which are among the most studied innate immune cells related to cancer progression. Moreover, SIRI was selected because it comprised three inflammatory markers, including lymphocytes, and is expected to complement the interactions between markers, including innate and adaptive immunity.

This study has several limitations. First, although we aimed to develop a recurrence prediction scoring system using multiple prognostic factors, the number of statistically significant variables was limited, preventing the construction of a valid model. Future studies with larger sample sizes are warranted to identify more reliable predictors.

Second, SIRI may have been influenced by external inflammatory conditions such as infections. To minimize this bias, we used preoperative test results alongside clinical assessments to exclude patients with signs of acute infection.

Third, key nutritional and body composition markers such as the Prognostic Nutritional Index (PNI) [60] and myosteatosis [61] were not included due to inconsistent data availability. However, accurate prognostic assessment in cancer patients often requires consideration of multiple factors beyond a single inflammatory marker. Nutritional status and muscle quality are known to be closely associated with systemic inflammation and clinical outcomes. Therefore, future prospective studies should incorporate such markers to enhance the robustness and precision of recurrence prediction.

Fourth, while SIRI demonstrated statistical significance, its individual discriminative power was limited (AUC = 0.565). A multi-marker model incorporating other inflammatory or nutritional indices (e.g., NLR, PLR, and PNI) may offer better predictive performance, but such analysis was not feasible in the current retrospective setting.

Lastly, the references cited in this study include several sources older than five years. This was not due to oversight but rather reflects the limited availability of recent literature addressing practical, inflammation-based prognostic markers. While molecular studies offer valuable mechanistic insights, their translation to routine clinical use remains limited. Our study aimed to bridge this gap by prioritizing accessible markers that can be easily applied in daily oncology practice.

## 5. Conclusions

Through this study, we confirmed that SIRI could be independently used as a risk factor for gastric cancer recurrence, regardless of the TNM stage. The fact that significant results were obtained after excluding the TNM stage (the strongest risk predictor) provides grounds for recognizing SIRI as an independent factor. In addition, a proportional relationship between SIRI values and the timing of gastric cancer recurrence was confirmed. This finding is a novel contribution that has not been previously reported. Since not only gastric cancer recurrence but also a disease-free survival period greatly affects the quality of life of patients, measuring SIRI as a predictive factor could have clinical significance. In addition, measuring SIRI values is a simple and cost-effective method, and the use of SIRI can be considered a tool to predict the prognosis of patients with gastric cancer undergoing postoperative adjuvant anticancer therapy. SIRI can be integrated into routine postoperative monitoring protocols for patients with stage II/III gastric cancer to provide early identification of individuals at higher recurrence risk. This allows for personalized follow-up schedules and timely intervention strategies, potentially improving patient outcomes. Furthermore, SIRI could be used to stratify patients in clinical trials, ensuring that those at higher recurrence risk receive more intensive monitoring and potentially novel therapeutic approaches. Although our findings suggest the clinical utility of SIRI as a prognostic marker, further research is necessary to confirm and expand upon these results. Future studies should focus on large-scale, multicenter studies to further validate the predictive value of SIRI across diverse patient populations. In addition, investigating the interaction between TNM staging and SIRI in greater detail, particularly in high-risk groups like stage III, may reveal insights into stage-specific prognostic value. Furthermore, exploring how SIRI can be incorporated into routine clinical follow-up schedules could enhance personalized patient care. Finally, combining SIRI with machine learning models that include multiple prognostic factors may enhance predictive accuracy, paving the way for more sophisticated risk stratification in patients with gastric cancer.

## Figures and Tables

**Table 1:** Baseline and clinicopathological characteristics of patients with gastric cancer who underwent gastrectomy.

		*n*	%
Age (years)	≤65	160	54.1
	>65	136	45.9
Sex	Male	202	68.2
	Female	94	31.8
Location	Cardia	21	7.1
	Body	135	45.6
	Antrum	134	45.3
	Whole	6	2
Differentiation	Well	3	1
	Moderate	155	52.7
	Poor	136	46.3
Depth of invasion	T1	24	8.1
	T2	50	16.9
	T3	115	38.9
	T4	107	36.1
LN metastasis	N0	53	17.9
	N1	72	24.3
	N2	83	28
	N3	88	29.7
TNM stage (8th)	Stage II	147	49.7
	Stage III	149	50.3

LN—lymph node; TNM—Tumor, Node, Metastasis.

**Table 2:** Chi-squared analysis of predictive factors of gastric cancer recurrence.

		Total, *n* (%)	No Recurrence, *n* (%)	Recurrence, *n* (%)	χ^2^ (*p*)
HER2	Negative	187 (100)	143 (77)	44 (23)	3.062 (0.08)
	Positive	83 (100)	55 (66)	28 (34)	
Lauren classification	Intestinal	152 (100)	108 (71)	44 (29)	0.353 (0.838)
	Diffuse	111 (100)	82 (74)	29 (26)	
	Mixed	28 (100)	21 (75)	7 (25)	
P53	Wild Type	69 (100)	55 (80)	14 (20)	1.541 (0.214)
	Overexpression	149 (100)	107 (72)	42 (28)	
SIRI	Low	267 (100)	202 (76)	65 (24)	15.345 * (<0.001)
	High	29 (100)	12 (41)	17 (59)	
EBV	Negative	92 (100)	74 (80)	18 (20)	0.136 (0.712)
	Positive	8 (100)	6 (75)	2 (25)	
E-cadherin	Not Decreased	125 (100)	90 (72)	35 (28)	2.601 (0.107)
	Decreased	41 (100)	24 (59)	17 (42)	
MSI	MSS/MSI-L	50 (100)	43 (86)	7 (14)	0.031 (0.860)
	MSI-H	6 (100)	5 (83)	1 (17)	
TNM stage	Stage II	147 (100)	130 (88)	17 (12)	37.97 * (<0.001)
	Stage III	149 (100)	84 (56)	65 (44)	

* *p* < 0.05. EBV—Epstein–Barr virus; MSI—microsatellite instability; MSS—microsatellite stability; MSI-L—MSI-low degree; MSI-H—MSI-high degree; SIRI—systemic inflammatory response index; TNM—Tumor, Node, Metastasis.

**Table 3:** Chi-squared analysis of gastric cancer recurrence according to the TNM stage.

		Total, *n* (%)	No Recurrence, *n* (%)	Recurrence, *n* (%)	χ^2^ (*p*)
TNM state II	Low SIRI	139 (100)	124 (90)	14 (10)	4.442 * (0.035)
	High SIRI	9 (100)	6 (67)	3 (33)	
TNM stage III	Low SIRI	129 (100)	78 (60)	51 (40)	6.535 * (0.011)
	High SIRI	20 (100)	6 (30)	14 (70)	

* *p* < 0.05; TNM—Tumor, Node, Metastasis; SIRI—systemic inflammatory response index.

## Data Availability

The datasets generated and analyzed during the current study are not publicly available because of the privacy of the research participants but are available from the corresponding author upon reasonable request.

## Abbreviations

The following abbreviations are used in this manuscript:

TNM staging	Tumor, Node, Metastasis staging
SIRI	Systemic inflammation response index
NLR	Neutrophil-to-lymphocyte ratio
PLR	Platelet-to-lymphocyte ratio
HER2	Human epidermal growth factor receptor 2
EBV	Epstein–Barr virus
MSI	Microsatellite instability
ROC	Receiver operating characteristic curve
CI	Confidence interval
PD-1/PD-L1	Programmed cell death protein 1/programmed cell death ligand 1
